# Investigating perturbed pathway modules from gene expression data via structural equation models

**DOI:** 10.1186/1471-2105-15-132

**Published:** 2014-05-06

**Authors:** Daniele Pepe, Mario Grassi

**Affiliations:** 1 Department of Brain and Behavioural Sciences, Medical and Genomic Statistics Unit, University of Pavia, Pavia, Italy

**Keywords:** Structural equation modeling (SEM), Model generation, Pathway analysis, Perturbed models, Perturbed edges

## Abstract

**Background:**

It is currently accepted that the perturbation of complex intracellular networks, rather than the dysregulation of a single gene, is the basis for phenotypical diversity. High-throughput gene expression data allow to investigate changes in gene expression profiles among different conditions. Recently, many efforts have been made to individuate which biological pathways are perturbed, given a list of differentially expressed genes (DEGs). In order to understand these mechanisms, it is necessary to unveil the variation of genes in relation to each other, considering the different phenotypes. In this paper, we illustrate a pipeline, based on Structural Equation Modeling (SEM) that allowed to investigate pathway modules, considering not only deregulated genes but also the connections between the perturbed ones.

**Results:**

The procedure was tested on microarray experiments relative to two neurological diseases: frontotemporal lobar degeneration with ubiquitinated inclusions (FTLD-U) and multiple sclerosis (MS). Starting from DEGs and dysregulated biological pathways, a model for each pathway was generated using databases information biological databases, in order to design how DEGs were connected in a causal structure. Successively, SEM analysis proved if pathways differ globally, between groups, and for specific path relationships. The results confirmed the importance of certain genes in the analyzed diseases, and unveiled which connections are modified among them.

**Conclusions:**

We propose a framework to perform differential gene expression analysis on microarray data based on SEM, which is able to: 1) find relevant genes and perturbed biological pathways, investigating putative sub-pathway models based on the concept of disease module; 2) test and improve the generated models; 3) detect a differential expression level of one gene, and differential connection between two genes. This could shed light, not only on the mechanisms affecting variations in gene expression, but also on the causes of gene-gene relationship modifications in diseased phenotypes.

## Background

Most of known diseases are complex diseases. This means that they are caused by the combination of genetic and environmental factors. The introduction of the concept of network biology [1] allowed the application of network based approaches for studying this type of diseases. These approaches rely on the possibility to represent molecules, as proteins or genes, as interaction networks. Microarray experiments of gene expression represent a useful tool to examine the change of gene expression profile in diseases. Many efforts were performed to build, starting from gene expression, molecular networks. This activity is sometimes referred to as reverse engineering of gene regulatory networks [2]. One type of method applied for this goal, relies on the Structural Equation Modeling (SEM), a general methodology used to address questions about complex systems [3]. SEM finds a number of applications in biological networks, for example in the inference of causal phenotype networks (see [4] for a review), genome-wide association studies (GWAS) and gene-environment interactions [5,6], as well as to measure effects of quantitative trait loci (QTLs) in linkage analyses [7-9]. The use of SEM in the analysis of microarray is not new. One of the first applications is shown by [10], who demonstrated that covariance structure analysis is a useful statistical method to find common transcriptional factors for a set of genes and to specify and evaluate hypothesized biological pathways. [11] applied SEM systematically for gene network reconstruction using gene expression data pre-processed with genetic algorithms. In most of the applications of SEM the aim is to infer networks starting from data. Thus, we can define these approaches as exploratory approaches. More recently, [12] described a confirmatory approach in microarray analysis.

In this paper we propose a SEM pipeline that, from initial and revised *a priori* network models, obtained by pathway analysis [13], is able to compare the path strengths between several groups and to determine the effect of factors analyzed on the paths. Our framework takes into consideration the generation of pathway models based on the principles of network theory such as the small network phenomena and the detection of modules [14]. Firstly considering how differentially expressed genes (DEGs) are connected by other genes in the microarray, we try to bring out which modifications in the gene network could be responsible of the differences observed between groups considered. Our approach relies on: 1) curated biological pathway databases, 2) the principles that characterize disease genes in biological networks, 3) grouping genes in Protein Information Resource (PIR) super-families [15] for facilitating the interpretation of the model. The proposed SEM pipeline is a combination of data-driven and knowledge-driven approaches. In fact, in order to generate perturbed pathway modules, we used curated biological pathways, representing the *a priori* biological knowledge about genes and their connections. The hardest part is to highlight which portion of the pathway is actually distinctive of the phenomenon being analyzed. We consider, as initial model, the one obtained from the shortest paths between every couple of DEGs. This process preserves the biological knowledge completely, as the connections in the new model are not inferred, but already present in the original pathway. The model is then fitted with SEM and improved by balancing between data-driven and knowledge-driven evidences obtained by the combination of SEM with the knowledge enclosed in public databases, relative to real and putative connections among genes. Finally, SEM with multiple group analyses supplies useful information to clinicians and biologists about experimental group differences, unveiling which connections and genes are statistically significant in the perturbed pathway models.

## Methods

### Illustration of the proposed pipeline

**Figure 1:**
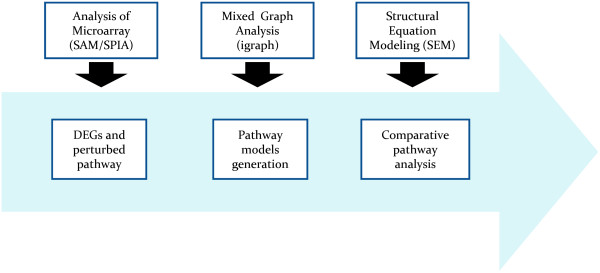
Pipeline proposed to generate and evaluate genetic pathway models.

The pipeline, similar to that described by [12], is illustrated in Figure 1. In step-1, DEGs were obtained by Significance Analysis of Microarray (SAM) [16] and the perturbed pathways by Signaling Pathway Impact Analysis (SPIA) [17] using KEGG database [18]; in step-2, the pathway models were generated by network analysis and evaluated with SEM in step-3 for: 1) improving the models generated by the biological pathways found; 2) testing if the pathway models differ across groups by multiple group analysis; 3) screening of single differences in expression (gene nodes) and in regulation (gene-gene edges) across groups. We used the implementation provided by the R packages samr [19] and SPIA [20] for SAM/SPIA procedures.

### Structural equations models (SEM)

SEM is a statistical procedure for confirmatory causal inference originated from path analysis proposed in 1921 by the American geneticist Sewall Wright [21]. It is based on multivariate linear regression equations, where the response variable in one regression equation may appear as a predictor in another equation. Indeed, variables may influence one-another reciprocally, either directly or through other variables as intermediaries. Additionally, correlated or uncorrelated unmeasured variables may indicate the presence of unobserved factors that influences observed variables.

In general, a SEM consists of a structural model describing (causal) relationships among latent (hidden) variables and a measurement model describing the relationships between the observed measurements and the underlying latent variables [22]. Here we consider SEM with observed variables only, and therefore no measurement models have been used [23]. Specifically, let *V* to be the index set of the *Y* observed variables, represented as the “parent” set {*pa*(*i*)|*i* ∈ *V*}, i.e. the explanatory variables of *Y*_*i*_, or as the “siblings” set {*sib*(*i*)|*i* ∈ *V*}, i.e. the unmeasured linked variables with *Y*_*i*_, respectively. These sets determine a system of linear equations:

$$Y_{i}=\sum_{j\in \text{pa}\left(i\right)}\beta_{\text{ij}}Y_{j}+U_{i}\;i\in V$$

and a covariance structure:

$$\mathrm{cov}\left(U_{i};U_{j}\right)=\left\{\begin{array}{ccc} \psi_{\text{ij}} & \mathrm{if}\;i=j & \text{or}\;j\in \text{sib}\left(i\right)\\ 0 & \text{otherwise} & \end{array}\right)$$

The system of linear equations affirms that every node is characterized by the relationships with his parents, while the covariance structure describes the relationships between unobserved nodes.

They encode two distinct causal assumptions: (1) a “weak” assumption on the possible existence of (direct) casual influences of explanatory variables on *Y*_*i*_, and (bi-directed) correlated unmeasured variables *U*_*i*_, quantified by the regression (path) coefficients *β*_*ij*_, and the covariances *ψ*_*ij*_, respectively; and 2) a “strong” assumption based on the absence of (direct) causal influences or (bi-directed) correlations of any observed/unobserved variables neither in the “parents” set pa(i) nor in the “siblings” set sib(i). In other terms, a weak assumption excludes some values for a parameter (the null value zero), but permits a range of other values; while, strong assumptions assume that parameters take specific values (null value zero or a fixed *a priori* value). The linear equations and the covariance structure can be encoded and visualized in a “path diagram”, that is, a mixed graph *G* = (*V*,*E*) featuring both directed (→) and bi-directed (↔) edges. The vertex set *V* includes the genes and the edge set *E* represents relations or reactions among vertexes. The “activity” of a given gene is embedded in a path diagram: the actions performed by a gene on downstream molecules, and the signals that it receives from upstream regulators. “Directed edges” between two genes (j→i, if and only if j ∈ pa(i)), measured by path coefficients (ranging usually from -1 to 1, if genes are standardized), represent expected change in the activity of the downstream gene, given a unit of change in the upstream gene while the values of the other genes remain constant. Considering that paths reflect a direct influence of one gene on another, negative path coefficients indicate ensemble inhibition (negative control) and positive paths measure net activation (positive control). “Bi-directed edges” between two genes (j↔i if and only if j ∈ sib(i), or equivalently, if and only if i ∈ sib(j)) encode a hidden common cause that may be interpreted as latent or unobserved measurement of upstream regulators that could account for the observed covariances (correlations) between the two genes.

One important feature of SEM is that direct and indirect effects can be computed and compared. “Directed paths” between two genes are the sequence of all the directed edges (j → k_1_,… → …, k_m_ → i) from genes *Y*_j_ to *Y*_i_. Each directed path is a channel along which information (gene’s activities) can flow, and so a “total effect” (TE) of gene *Y*_j_ on gene *Y*_i_ is defined as the total sum of the products of the sequence of arrows (edges) along all directed paths from *Y*_j_ to *Y*_i_. Accordingly, a “indirect effect” (IE) of gene *Y*_j_ on gene *Y*_i_ represents the portion of the total effects not considering the directed edge effect (DE), i.e. TE = DE + IE.

The well-known SEM analysis consists of four steps [22]: a) definition and identification of an initial path model, b) estimation of parameters, c) evaluation of the fitting, and d) model modification.

### Initial model building

Specification of initial pathway models (step a) was obtained taking the perturbed pathways and converting them in directed graphs or gene networks. Generalizing, each pathway can be seen as a mixed graph. The idea is to understand how DEGs are connected in the perturbed pathways by other microarray genes. A natural way to solve this problem is to identify the shortest paths (geodesic distance) between DEGs. The geodesic distance d_geo_(*y*_i_, *y*_j_) between two DEGs, *y*_i_ and *y*_j_, is defined as the minimum distance between these two genes. The function *get.shortest.paths*( ) of the R package igraph was used to compute all the shortest paths [24]. Define the microarray genes, DEGs, and not DEGs in the following way: *MG =* {*mg*_*1*_*, mg*_*2*_*, …, mg*_*m*_}; *DEG =* {*deg*_*1*_*, deg*_*2*_* ,…, deg*_*n*_} and *NDEG =* {*ndeg*_*1*_*, ndeg*_*2*_*,…,ndeg*_*m-n*_}, where *MG = DEG* ∪ *NDEG* and *DEG* ∩ *NDEG =* {∅}. Each shortest path could be represented as a list of nodes Y_*k*_*=* (*y*_*i*_*, y*_*i+1*_* ,..., y*_*j-1,*_*y*_*j*_) and a list of the corresponding edges *E*_k_ = (e_i(i+1)_, ..., e_(j-1)j_) where (*y*_*i*_*, y*_*j*_) ∈ *DEG*; (*y*_*i+1*_* ,…, y*_*j-1*_) ∈ (*DEG* ∨ *NDEG*); Y_k_ ⊆ Y and *E*_k_ ⊆ *E*. The shortest paths for each pathway constitute *k* (*k* = 1, …, *K*) subgraphs *G*_k_ = {*Y*_k_,*E*_k_} of the original pathway, *G* = {*Y*, *E*}. Not all DEGs and NDEGs will be included in the shortest paths. Therefore, we define two new sets: *DEG*(*s*) and *NDEG*(*s*) respectively, the sets of DEGs and the set of not DEGs that include all genes in shortest paths, where *DEG*(*s*) ⊆ *DEG* and *NDEG*(*s*) ⊆ *NDEG.*

To reach the final model the *NDEG*(*s*) that connect *DEG*(*s*) are grouped in basis to their PIR superfamily (PIRSF). Based on the evolutionary relationships of whole proteins, this classification system allows annotation of both specific biological and generic biochemical functions. The PIRSF can be represented as *SUPF =* {*supf*_*1*_*, supf*_*2*_*,…, supf*_*g*_}, where ∀ *supf*_*i*_ ⊆ *NDEG*(*s*) and ∀_i≠j_ *supf*_*i*_ ∩ *supf*_*j*_ *=* {∅}. Using this information, each original shorthest path *G*_k_ = {*Y*_k_,*E*_k_} is transformed in a new shortest paths, G*_k_ = {Y*_k_ = (y*_i_, y*_i+1_,…, y*_j-1,_ y*_j_), E*_k_ = (e*_i(i+1)_,…, e*_(j-1)j_) }, where (y*_i+1_,…, y*_j-1_) ∈ (*DEG(s)* ∨ *SUPF* ∨ *NDEG(s)*) and E* ⊆ E. The function to obtain the final graph, G* = (Y*,E*), is described in the following pseudo-code:

The graph G* = (Y*,E*) is the fusion of all shortest paths found, where each node and each edge cannot be present more than once, the self-loops are not considered but the feed-backs and cycles were preserved. To ensure the identification of the initial models, the “block-recursive” criterion of Rigdon [25] and the “bow free” criterion of Brito and Pearl [26] were applied. The first affirms that reciprocal relationships, feedback loops, or covariances are segregated into groups, or *blocks*, with no more than two equations per block. The second affirms that a model is ensured if variables standing in direct causal relationships (directed edges) do not have correlated errors (bi-directed edges). So a new graph is attained in which the DEGs are connected by other DEGs, PIRSFs or NDEGs. In this way a model was created for each significant pathway found.

Successively, PIRSFs composite variables are defined considering not DEGs, present in shortest paths, as causal indicators of latent (hidden) constructs [27]. To generate the PIRSFs, a principal component analysis (PCA) was performed on genes belonging to a PIRSF and the principal component scores of the first principal component (PC1) were considered as the values that characterize the PIRSF. Only PIRSFs for which the PC1 represents 50% or more of the total variance are considered. At the end of process we have the initial SEM model.

The pathway graph conversion, the graph analysis, and the PC1 scores are obtained by graphite [28], igraph [24] and stats [29] R packages, respectively, while R functions for network analysis are implemented ad hoc, and are available Additional file 1.

### SEM fitting

For parameter estimation (step-b), the classic derivation of the Maximum Likelihood estimation (MLE) is used, that assumes all observed variables are jointly Gaussian. The system of structural equations and covariance structure of unmeasured variables can be written compactly in a matrix form as: *Y* = *BY + U*, and Cov(*U*) = Ψ. This specification induces a structure on the covariance matrix of the joint distribution of the genes Y as:

$$\Sigma \left(\theta \right)={\left(I-B\right)}^{-1}\Psi {\left(I-B\right)}^{-T}$$

where *θ=*(*β*; *ψ*) is the list of the free parameters in the model of dimension *t.* The unknown parameters are estimated so that the implied covariance matrix Σ(*θ*) is close to the observed sample covariance matrix *S*.

The assessment of the model (step-c) involves the Likelihood Ratio test (LRT) converted to a Chi-square test of the fitted model. Specifically, let Σ_0_ = E(*S*) to be the true population covariance matrix, and Σ(θ) the model-implied covariance matrix. The hypothesis to be tested is:

$$H_{0}:\Sigma_{0}=\Sigma \left(\theta \right)\;\text{vs}.\;H_{1}:\Sigma_{0}\neq \Sigma \left(\theta \right)$$

The chi-square test is then χ^2^ = -2logLRT = -2[logL(Σ(*θ*)) - logL(Σ_0_)] with *d* = *p*(*p* + 1)/2-*t* degree of freedom (d.f.). logL( ) represents the log-likelihood of the model, *p* the number of genes, *t* the number of parameters of the fitted model. Not-significant *P*-values (*P* >0.05) indicate that the model provides a good fit to the data. The *P*-values are derived by using the χ^2^(*d*) distribution or a resampling bootstrap distribution [30].

An alternative procedure [31] assumes that in the population, a model-implied covariance matrix Σ(*θ*_0_), which is approximately correct, is in the neighborhood of Σ_0_. So the null hypothesis of “exact fit” is replaced by the null hypothesis of “close fit”:

$$H_{0}:\Sigma \left(\theta_{0}\right)-\Sigma \left(\theta \right)<\varepsilon =0.05\;\mathrm{vs}.\;H_{1}:\varepsilon >0.05$$

and the Root Mean Square Error of Approximation (RMSEA) measures the discrepancy *ε* for the fitted model:

$$\text{RMSEA}=\sqrt{\max\left(0;\chi^{2}-d\right)/d\left(n-1\right)}$$

*P*-values for RMSEA are set up from the non-central χ^2^(*λ*,*d*) distribution with non-centrality parameter, *λ* = (*n*-1) × *d* × 0.05^2^ or from a resampling bootstrap distribution. The null hypothesis of close fit is not rejected if *P* > 0.05.

We also consider the Standardized Root-Mean-square Residual (SRMR), one of the most used SEM fit indices. SRMR is a measure based on the differences between observed (*s*) values and the ones obtained from the model (σ) of the covariance matrix:

$$\text{SRMR}=\frac{\sum_{j=1}^{p}\sum_{k=j+1}^{p}{\left(s_{\text{jk}}-\sigma_{\text{jk}}\right)}^{2}/s_{\text{jj}}s_{\text{kk}}}{p\left(p+1\right)/2}$$

SRMR values <0.10 are assumed as an adequate fitting measure, whereas values <0.05 may be considered as a good fit [32].

Finally, the model refinement (step-d) is obtained adding new directed or bi-directed edges to the initial model. This modification was needed considering that the initial model is only a simplified representation of the whole pathway. The criteria used for the refinement are based on the combination of three elements. First, the modification indexes (MI), that is an estimate of the decrease in the χ^2^-score statistic that would result by freeing each fixed (=0) parameter in the model; second, z-tests (=parameter estimate/standard error) of the MLE; and finally, biological evidences obtained by STRING database [33] and by the existence of a direct path between the nodes that MI proposes to connect. The following heuristic stepwise strategy was used:

Heuristic stepwise procedure:

Input: list of the fixed (=0) parameters (paths and/or covariances) in the model.

Output: new free parameters (paths and/or covariances) in the model.

Steps:

1. freeing just a single parameter (path coefficient or covariance) at a time, and these in turn are sorted in descending order of magnitude using MI;

2. verify if the edge (path coefficient or covariance) to add is present in STRING or when the edge is a path coefficient, if it represents a direct path that connects the nodes in the pathway selected, and then add this new edge in the model;

3. fit the model and if the new edge is statistically not significant (*P* > 0.05, one-sided), using a z value (z<|1.64|), remove it and repeat step 1-2;

4. STOP the selection procedure if the model achieves a non significant LRT (*P* > 0.05) or RMSEA (*P* of “close” fit > 0.05) or SRMR < 0.1, otherwise repeat step 1-3

### Multiple-group analysis

When data are observed from multiple subsamples, the representation of groups with “indicator variables”, considered as nodes, allows to recognize DEGs. Instead, “multiple-group analysis” allows to identify differentially regulated genes (DRGs) across groups.

Specifically, define μ_1_(θ) and Σ_1_(θ) as the model-implied mean vector and covariance matrix of group 1 (experimental group) respectively and μ_2_(θ) and Σ_2_(θ) as the corresponding moments of group 2 (control group). For each models, two omnibus tests are performed considering the two experimental conditions (groups), one for the differential expression genes (nodes) and the other for the strength of the edges. In the first case, the hypothesis to be tested is:

$$H_{0}:\mu_{1}\left(\theta \right)=\mu_{2}\left(\theta \right)\;\mathrm{vs}.\;H_{1}:\mu_{1}\left(\theta \right)\neq \mu_{2}\left(\theta \right)$$

while, in the second case, is:

$$H_{0}:\Sigma_{1}\left(\theta \right)=\Sigma_{2}\left(\theta \right)\;\text{vs}.\;H_{1}:\Sigma_{1}\left(\theta \right)\neq \Sigma_{2}\left(\theta \right)$$

In the “null” model (*H*_0_), the mean or covariance estimates are constrained to be equal across groups; in the “alternative” model (*H*_1_), they are allowed to differ across groups. The statistical significance is determined by comparison of LRT chi-square (χ^2^diff) values at a given degree of freedom (d.f. diff). If there is a significant difference (*P* < 0.05) in the chi-squared goodness-of-fit index, the groups differ significantly for one or more specific gene expression (nodes) and/or gene-relationships (edges). Finally, three path-coefficient differences are screened: 1) “up/down” expression (gene nodes), testing the “zero value” for the group indicator variable (C = experimental =1, and C = control = 0) path coefficients; 2) “up/down” regulation (gene edges), testing the “zero value” for the differences of path coefficients across groups; 3) “on/off” regulation (gene edges) with respect to *a priori* KEGG gene regulation target, testing the “zero value” of the edge coefficients across groups.

Specifically, assume C to be the path coefficient matrix of the group indicator variables and *c*_*i*_ be an element of the matrix C. Let B_1_ and B_2_ to be the corresponding path coefficient matrices in the experimental and control groups; D = B_1_ – B_2_ and *d*_*ij*_ be an element of the matrix D. We consider the test statistics:

$$\begin{array}{l} t_{C}=c_{i}/\text{SE}\left(c_{i}\right)\;\text{and}\;t_{D}=d_{\text{ij}}/\text{SE}(d_{\text{ij}})\\ t_{1}=b_{\text{ij}1}/\text{SE}\left(b_{\text{ij}1}\right)\;\text{and}\;t_{2}=b_{\text{ij}12}/\text{SE}(b_{\text{ij}2}) \end{array}$$

where *SE*( ) is the estimated standard error of the parameters. The statistic *t*_C_ can be used to test the conditional “up/down” expression level difference of one gene between groups, given the parents of the gene in the network. Similarly, *t*_*D*_ checks the conditional “up/down” expression regulatory differences of one gene on another between groups. Moreover, *t*_1_ and *t*_2_ check the “on/off” regulatory differences compared to a priori KEGG pathway. The *P*-values of these statistics (two-sided, for *t*_C_ and *t*_D_ and one-sided, for *t*_1_ and *t*_2_) are derived either asymptotically from the *N*(0,1)-distribution or empirically from the nonparametric-based or using model-based bootstrap distribution with B bootstrap samples (usually, B = 100, or 1000).

Note that the marginal bivariate test of DEGs with SAM approach can be regarded as the special case of the conditional test with *t*_C,_ when the pathway graph is G = (Y, ∅), so pa(y) = ∅ for all genes ∈Y.

We use the implementation provided by the lavaan [34] R package for estimation, evaluation, and modification of SEM data analysis, and R codes is available in Additional file 1.

## Results

The above described method was applied to two gene expression microarrays datasets, one from a study on FTLD-U and the other on MS.

### FTLD-U analysis

The data were obtained from a microarray experiment that analyzes various brain regions of patients affected by FTLD-U in presence of the mutation in the progranulin gene. Two groups were selected: one affected by FTLD with mutation in the progranulin gene (15 samples) and the other constituted by the control (17 samples). Data are freely available at Gene Expression Omnibus (GEO) database with ID GSE13162. For our analysis, we used normalized expression values submitted in the database. In the first step, SAM was performed using a delta value of 1.03 and a minimum fold-change of 2. The number of genes up-expressed was 207 while the number of gene down-expressed 244. Using this list of DEGs, the SPIA analysis found seven important pathways for the explanation of the role of the progranulin mutation on the FTLD-U, as showed in the Table 1.

**Table 1:** Perturbed pathways obtained by SPIA on FTLD-U data

**Name pathway**	**pSize**	**NDE**	**pNDE**	**tA**	**pPERT**	**pGFdr**	**Status**
Glutamatergic synapse	77	11	0.000	-6.557	0.064	0.006	Inhibited
GABAergic synapse	60	10	0.000	0.632	0.804	0.017	Activated
Calcium signaling pathway	166	17	0.000	0.072	0.993	0.021	Activated
Amphetamine addiction	55	8	0.001	-2.685	0.457	0.047	Inhibited
Gap junction	85	10	0.001	5.216	0.454	0.047	Activated
MAPK signaling pathway	235	18	0.001	-5.802	0.253	0.047	Inhibited
ECM-receptor interaction	82	7	0.022	6.150	0.015	0.047	Activated

pSize = number of genes in the pathway; NDE = number of DEGs in the pathway; pNDE = p-value of the enrichment; tA = total perturbation; pPERT = p-value of the perturbation; Status = direction of the perturbation.

The most of the dysregulated pathways, as the MPAK signalling pathway, the calcium signalling pathway, the gap junction and the ECM-receptor interaction, confirm the analysis of [35]. The dysregulated pathways with a significant p-PERT were the glutamatergic synapse and GABAergic synapse. The role of the glutamate in the acute and neurodegenerative processes were well described in literature [35-38]. Meldrum [36] illustrated three different pathological mechanisms of action of the glutamate in the neurodegeneration. Glutamate can be neurotoxic through an agonist effect on the N-methyl-D-aspartate (NMDA), α-amino-hydroxy-5-methyl-4-isoaxaleproprionicacid (AMPA), kainate or Group I metabotropic receptors. The relative contribution of these different classes of receptor*s* vary according to the neurons involved and a variety of other circumstances. Selective neuronal death subsequent to the epileptics status appears to be highly dependent on NMDA receptor activation. Acute neuronal degeneration after transient global or focal cerebral ischemia seems to be dependent on both NMDA and AMPA receptors. Regarding the GABAergic pathway, a loss of glutamatergic pyramidal cells and calbindin-D28k-immunoreactive GABAergic neurons in the frontal and temporal cortices of patients with FTLD [39] and FTLD with motor neuron disease [40] was reported.

### SEM analysis of glutamatergic synapse KEGG pathway

For sake of brevity, model specification was obtained only for the glutamatergic synapses pathway, but the same procedure could be used for all significant pathways. We started from the original KEGG pathway and then, passing through the shortest paths model, we reached the PIRSF model. Four composite variables (PCA1) were generated: 1) PLC-beta (PIRSF000956) by entrez ID genes 5330, 5331, 23236: variance explained 66%; 2) adenylate cyclase (PIRSF001445) by entrez ID genes 113, 114, 155: variance explained 68%; 3) GTP-binding regulatory protein Gs alpha chain (PIRSF002400) by entrez ID genes 2776, 2778: variance explained 53%; 4) ionotropic glutamate receptor (PIRSF002437) by entrez ID genes 2902, 2903, 2905: variance explained 54%. The graph reduction of the model specification process was the following: from KEGG (mean degree = 11.431, number nodes(edges) = 92 (526)) to DEGs shortest paths model (mean degree = 3.769 , number nodes(edges) = 26 (49)), and to PIRSF model (mean degree = 2.947, number nodes(edges) = 19 (28)). The initial fitting indices were poor (χ^2^ (df) = 600.1 (25), *P* < 0.001, RMSEA (P-close) =0.320 (<0.001), SRMR = 0.450). This is likely because the existing pathway databases do not even contain all pathway information presented in the public literature [41], and because the model generated was a simplification of the real connections between nodes. Twenty-two directed edges were added, six using STRING database and sixteen using graph information. The final pathway model was an adequate approximation of the observed covariance matrix, as demonstrated by the SRMR index (0.092). Two-group analysis of the final pathway model revealed a significant global mean (χ^2^ diff(df) = 48.5 (19), *P* < 0.001 of H_0_: μ_1_ = μ_2_ subject to Σ_1_ = Σ_2_) and covariance differences (χ^2^ diff (df) = 110.9 (51), *P* < 0.001 of H_0_: Σ_1_ = Σ_2_ subject to μ_1_≠ μ_2_). The specific tests that consider the effect of the progranulin mutation on every gene and every edge are summarized in Tables 2 and 3.

**Table 2:** Single node and edge differences found between FTLD-U with progranulin mutation and control groups

**Path**	**Type**	**FTLD-U Progranulin (P)**	**Control (C)**	**Difference (95% CI)**	**P-value**	**up/down**
1742 ← group	Group on node	-6.024	-5.540	-0.484(-0.87; -0.10)	0.014	P down-expressed
5532 ← group	Group on node	9.606	10.706	-1.101 (-1.64; -0.56)	0.000	P down-expressed
2785 ← group	Group on node	8.453	9.734	-1.281 (-1.95; -0.62)	0.000	P down-expressed
5534 ← group	Group on node	7.233	8.308	-1.075 (-1.65; -0.51)	0.000	P down-expressed
gtp_bind ← group	Group on node	2.374	2.626	-0.252 (-0.44; -0.06)	0.000	P down-expressed
2911 < - > 9454	Binding/association	0.178	0.813	-0.635 (-1.27; -0.00)	0.049	P down-regulated
3708 < -9456	Binding/association	0.457	-0.205	0.662 (0.34; 0.98)	0.000	P up-regulated
ade_cycl < -gtp_bind	activation	-2.044	-0.840	-1.204 (-2.28; -0.13)	0.028	P down-regulated
5613 < -107	Indirect	-0.012	-0.324	0.312 (0.02; 0.60)	0.034	P up-regulated
5613 < -ade_cycl	Indirect	0.446	-0.413	0.858(0.43; 1.20)	0.000	P up-regulated
5579 < -plc_b	Indirect	3.677	1.110	2.567(0.86; 4.28)	0.003	P up-regulated
22941 < -1742	String	1.306	0.570	0.736(0.12; 1.35)	0.002	P up-regulated
plc_b < -9229	Directed path	0.262	0.558	-0.297(-0.56; -0.03)	0.028	P down-regulated
ade_cycl < -5532	Directed path	-0.463	0.208	-0.671(-1.16; -0.18)	0.007	P down-regulated
ade_cycl < -5534	Directed path	-0.713	-0.168	-0.545(-1.05 -0.05)	0.032	P down-regulated
5613 < -22941	Directed path	-0.581	-0.039	-0.542(-0.95; -0.13)	0.010	P down-regulated
22941 < -glutam_recp	Directed path	0.044	-0.278	0.322(0.09; 0.55)	0.006	P up-regulated
9455 < -glutam_recp	Directed path	-0.128	-0.462	0.334(0.04; 0.92)	0.024	P up-regulated

glutam_recp = PIRSF ionotropic glutamate receptor; ade_cycl = PIRSF adenylate cyclise; plc_b = PIRSF PLC-beta; gtp_bind = PIRSF GTP-binding regulatory protein Gs alpha chain.

**Table 3:** Not significant (null edge) in FTLD-U with progranulin mutation and in control groups

		**Progranulin (P)**		**Control (C)**		
**Paths**	**Type**	**Estimate**	**P-value**	**Estimate**	**P-value**	**P/C**
2911 < -5534	Activation	-0.024	0.238	0.232	0.927	OFF/OFF
gtp_bind < -2911	Activation	-0.049	0.167	-0.138	0.500	OFF/OFF
1742 < -glutam_recp	Binding/association	-0.192	0.008	-0.376	0.200	ON/OFF
50944 < -9229	Binding/association	0.131	0.138	0.160	0.282	OFF/OFF
22941 < -9229	Binding/association	-0.245	0.811	-0.034	0.276	OFF/OFF
9456 < -22941	Binding/association	-0.114	0.632	0.130	0.553	OFF/OFF
9456 < -50944	Binding/association	0.742	0.133	0.931	0.098	OFF/OFF
9455 < -22941	Binding/association	0.142	0.224	-0.407	0.042	OFF/ON
9454 < -50944	Binding/association	0.519	0.466	0.226	0.358	OFF/OFF
3708 < -9455	Binding/association	-0.322	0.067	-0.137	0.432	OFF/OFF
3708 < -9454	Binding/association	0.462	0.055	0.216	0.001	OFF/ON
107 < -gtp_bind	Activation	0.198	0.525	0.136	0.743	OFF/OFF
plc_b < -gtp_bind	Activation	-0.275	0.225	-0.234	0.127	OFF/OFF
5613 < -107	Indirect	-0.012	0.000	-0.324	0.926	ON/OFF
1742 < -50944	String	0.971	0.013	1.166	0.062	ON/OFF
3708 < -2911	String	-0.068	0.054	0.143	0.501	OFF/OFF
9229 < -50944	String	-0.490	0.116	-1.755	0.502	OFF/OFF
ade_cycl < -5532	Directed path	-0.463	0.169	0.208	0.022	OFF/ON
ade_cycl < -5534	Directed path	-0.713	0.107	-0.168	0.003	OFF/ON
glutam_recp < -5532	Directed path	-0.531	0.000	-1.017	0.061	ON/OFF
5579 < -9456	Directed path	0.228	0.000	0.654	0.470	ON/OFF
5579 < -5534	Directed path	0.295	0.000	0.986	0.388	ON/OFF
5613 < -22941	Directed path	-0.581	0.741	-0.039	0.001	OFF/ON
22941 < -glutam_recp	Directed path	0.044	0.000	-0.278	0.696	ON/OFF
3708 < -22941	Directed path	0.264	0.000	0.443	0.072	ON/OFF
1742 < -9229	String	-0.626	0.027	-1.399	0.126	ON/OFF

glutam_recp = PIRSF ionotropic glutamate receptor; ade_cycl = PIRSF adenylate cyclise; plc_b = PIRSF PLC-beta; gtp_bind = PIRSF GTP-binding regulatory protein Gs alpha chain.

Four genes and one PIRSF of the glutamatergic model resulted influenced by the group: genes 1742 or PSD-95, 5532 or PPP3CB, 2785 or GNG3, 5534 or Ppp3r1 and the PIRSF of the glutamate receptor. An important role could be played by PSD-95 gene that is believed to be involved in the synapse maturation, in the induction of a network of neurotransmitter receptors, scaffolding proteins and ionotropic glutamate receptors [42]. The gene PPP3CB and the gene GNG3 are associated to the Wnt signalling correlated to the dysregulation in the case of progranulin deficiency [43]. To note that four of the nodes influenced by the group were involved in the perturbed edges described in the Table 1. Giving a look to the significant edges found, the relationships 22941 <-1742 and 22941 <-PIRSF “glutamate receptors” are well note in the literature. In fact, the gene 229141 or SHANK2 plays a critical role both in the integration of the various postsynaptic membrane proteins, cell-adhesion molecules, signal components, scaffolding proteins, and actin-based cytoskeleton, part of the PSD protein network (activated by the PSD-95) [44], and in the organization of the glutamate receptors [45]. The edges 3708 <-9456 that involves the gene Itpr1 and the gene HOMER1 were very interesting. The relationships are involved in the spinocerebellar ataxia in human as described by [46]. Also other links that include the PIRSF adenylate cyclase, GTP-binding regulatory protein and the PLC-beta could be useful to interpret the role of mutation in the progranulin gene group.

**Figure 2:**
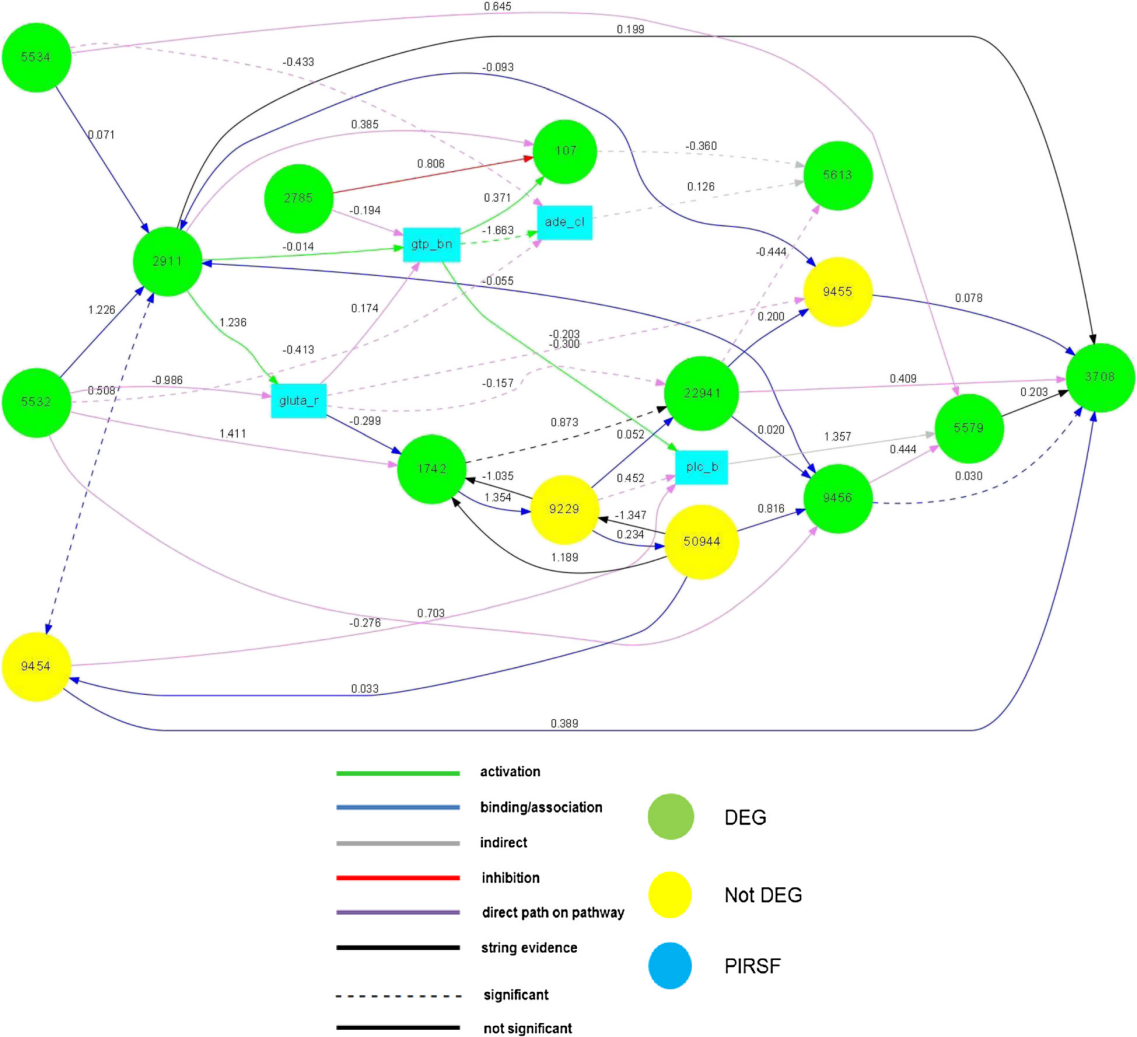
**Final model of the Glutamatergic synapse pathway.** The graph displays which nodes and edges are different between the yes/no mutant groups. The nodes (with “Entrez” name) or edges are mapped with different colours according to up/down expression or regulation, respectively.

The comparison with KEGG edges revealed that 17 edges were not active (off) in the “mutant” group and 21 in the “control”. In contrast the edges activated (on) was 9 and 5 in mutant and control, respectively (cf. Table 3). The Figure 2 illustrates the model and the links found statistically significant between the yes/no mutant groups.

### Multiple sclerosis

MS is a neurodegenerative disease with a presumed autoimmune component. The genome-wide expression study in peripheral blood mononuclear cells (PBMC), from 12 MS patients and 15 controls, was performed in order to identify DEGs and dysregulated pathways. Data are freely available at GEO database with ID GSE21942. For the analysis, we used expression values submitted in the database. The SAM was performed using a delta value of 0.95 and a minimum fold-change of 2. The number of genes up-expressed was of 133 while the number of gene down-expressed of 92. Using this list of DEGs, the SPIA analysis found three important pathways that could be involved in the mechanism of the development of the MS, as showed in the Table 4.

**Table 4:** Perturbed pathways obtained by SPIA on MS data

**Name pathway**	**pSize**	**NDE**	**pNDE**	**tA**	**pPERT**	**pGFdr**	**Status**
B cell receptor signaling pathway	73	8	0.000	6.822	0.372	0.000	Activated
Fc gamma R-mediated phagocytosis	89	4	0.015	-13.66	0.006	0.036	Inhibited
Salmonella infection	71	5	0.001	-4.901	0.174	0.041	Inhibited

pSize = number of genes in the pathway; NDE = number of DEGs in the pathway; pNDE = p-value of the enrichment; tA = total perturbation; pPERT = p-value of the perturbation; Status = direction of the perturbation.

The first, B cell receptor (BCR) signaling pathway, is an important component of adaptive immunity. B cells produce and secrete millions of different antibody molecules, each of which recognizes a different antigen. This signalling ultimately results in the expression of immediate early genes that further activate the expression of other genes involved in B cell proliferation, differentiation and immunoglobulin (Ig) production as well as other processes. The role of B cells is well known in MS [47,48]. The second one, Fc gamma R-mediated phagocytosis pathway, includes specialized cell types as macrophages, neutrophils, and monocytes that take part in host-defence mechanisms. Expression of the inhibitory Fc gamma receptor IIB (FcγRIIB) plays an important role during peripheral B cell development, which prevents memory B cells with low affinity or self-reactive receptors from entering the germinal center and becoming IgG positive plasma cells [49]. Furthermore, the decreased expression of Fc gamma RIIB or non-functional Fc gamma RIIB variants are consistently associated with the development of autoimmune tissue inflammation [49]– [51]. Considering the connection between the Fc gamma R-mediated phagocytosis and B cells, and the association of this pathway with autoimmune inflammation, we could conclude that also this pathway could be implicated in the MS phenotype.

Salmonella infection, the third pathway, may appear less interesting, nevertheless it is connected with response to infections that, as showed for the two previous pathways, plays an important role in MS.

### SEM analysis of Fc gamma R-mediated phagocytosis KEGG

As an example, we analyzed the Fc gamma R-mediated phagocytosis pathway. The model was obtained starting from the KEGG pathway and finding the shortest paths between DEGs (no PIRSF reduction was performed). The graph reduction of the model specification was from KEGG pathway (mean degree = 10.404, number nodes(edges) = 94 (489)) to DEGs shortest paths model (mean degree = 3.647, number nodes(edges) = 17(31)). The initial model had a poor fit (χ^2^ (df) = 339.410 (105), *P* < 0.001, RMSEA (*P*-close) =0.288 (<0.001), SRMR = 0.321) and the final model was adequate considering the SRMR index (0.098) and RMSEA (*P*-close) 0.111 (0.059). To reach the final model, twenty edges were added: fifteen using as driving criteria the presence of a directed path between the nodes in the original pathway, and five using STRING database information.

Two-group analysis of the final pathway-model revealed a significant global mean (χ^2^ diff(df) = 40.3 (16), *P* < 0.001 of H_0_: μ_1_ = μ_2_ subject to Σ_1_ = Σ_2_) and covariance differences (χ^2^ diff (df) = 124.4 (48), *P* < 0.001 of H_0_: Σ_1_ = Σ_2_ subject to μ_1_≠ μ_2_).

The specific tests, that analyze the effect of the disease-model on genes and edges, are reported in Tables 5 and 6. Five genes resulted group-sensitive: the genes 382 or ARF6, 10093 or ARPC4, 5580 or PRKCD, 5321 or plag2g4a, 1399 or CRKL. The gene ARF6, as showed by [52], could be implicated in the disruptive effects of IL-1b, a gene recently associated with MS [53]. In addition, other group sensitive genes could be associated with MS [54,55]. Considering the edges perturbed, the links between the gene 382 or ARF6 and the gene 1399 or CRKL resulted very interesting. The IL-1 beta, as reported [52], has an effect on the endothelial stability by the cascade MYD88 – ARNO and ARF6, a known regulator of adherents protein localization. In turn the gene CRKL, an adapter protein required for the spreading of epithelial colonies and breakdown of epithelial colonies and the breakdown of adherents junctions in response to hepatocyte growth factor, modulates the gene ARF6 [56]. So the genes IL-1b, ARF6 and CRKL could be involved in the same pathological mechanism. Other interesting significant edges were the connections between gene 23396 or PIP5K1C with gene 8612 or Ppap2c and with gene 8613 or Ppap2b. The gene PIP5K1C catalyzes the synthesis of phosphatidylinositol 4,5-bisphosphate, an essential lipid molecule in various cellular processes. As described by [57] the phosphatidylinositol-4, 5-bisphosphate synthesis has a critical role in the regulation of multiple steps of the synaptic vesicle cycle. This gene is connected with Ppap2c and Ppap2b, genes that have a role in metabolic pathways controlling the synthesis of glycerophospholipids and triacylglycerols, and in receptor-activated signal transduction mediated by phospholipase D, considered a susceptibility factor in sclerosis [58].

**Table 5:** Single node and edge differences found between MS and control groups

**Path**	**Type**	**Multiple sclerosis ( MS )**	**Control (C)**	**Difference (95% CI)**	**P-value**	**Up/down**
382 ← group	Group on node	-9.002	-7.963	-1.04 (-1.65; -0.43)	0.001	MS down-expressed
10093 ← group	Group on node	-0.452	0.032	-0.48 (-0.93; -0.04)	0.033	MS down-expressed
5580 ← group	Group on node	-3.054	-2.648	-0.41 (-0.70; -0.11)	0.007	MS down-expressed
5321 ← group	Group on node	0.694	1.240	-0.55 (-1.00;-0.09)	0.020	MS down-expressed
1399 ← group	Group on node	4.688	6.126	-1.44 (-1.82;-1.05)	0.000	MS down-expressed
8613 **↔** 8612	Indirect	0.001	-0.002	0.003 (0.001;0.004)	0.038	MS up-associated
23396 ← 8613	Indirect	0.643	-4.981	5.62 (2.44; 8.81)	0.001	MS up-regulated
23396 ← 5880	Activation	0.079	1.207	-1.13 (-1.91;-0.35)	0.005	MS down-regulated
23396 ← 8612	Indirect	-6.219	-0.945	-5.27 (-6.62; -3.92)	0.000	MS down-regulated
10093 ← 8936	Activation	0.576	0.267	0.31 (0.08; 0.53)	0.007	MS up-regulated
5581 ← 8613	Indirect	0.002	0.953	-0.95 (-1.41; -0.49)	0.000	MS down-regulated
5581 ← 8612	Indirect	1.343	0.104	1.24 (0.78; 1.70)	0.000	MS up-regulated
382 ← 1399	Directed path	0.970	0.144	0.83 (0.19; 1.46)	0.011	MS up-regulated
7454 ← 5338	Directed path	1.070	0.111	0.96 (0.28; 1.64)	0.006	MS up-regulated
5604 ← 8613	Directed path	0.618	3.133	-2.52 (-4.64; -0.39)	0.020	MS down-regulated
23396 ← 1399	Directed path	-0.323	-0.755	0.43 (0.24; 0.62)	0.000	MS up-regulated

**Table 6:** Not significant (null edge) in MS and in control groups

		**Multiple sclerosis (MS)**		**Control (C)**		
**Paths**	**Type**	**Estimate**	**P-value**	**Estimate**	**P-value**	**MS/C**
23396 ← 382	Activation	0.114	0.657	0.082	0.324	OFF/OFF
10093 ← 7454	Activation	0.419	0.199	0.065	0.726	OFF/OFF
5580 ← 8612	Indirect	0.326	0.388	5.119	0.144	OFF/OFF
5894 ← 5580	Activation	0.338	0.207	-0.103	0.657	OFF/OFF
5894 ← 5581	Activation	1.399	0.436	-2.815	0.349	OFF/OFF
5595 ← 5604	Phoshorylation	-0.107	0.808	-0.963	0.408	OFF/OFF
1794 ← 1399	Binding/association	0.056	0.399	-0.007	0.747	OFF/OFF
8936 ← 5880	Activation	0.015	0.972	-0.410	0.240	OFF/OFF
7454 ← 382	Directed path	0.057	0.753	-0.076	0.696	OFF/OFF
5604 ← 5338	Directed path	-0.024	0.810	-0.170	0.454	OFF/OFF
5595 ← 5338	Directed path	0.124	0.404	0.590	0.065	OFF/OFF
5338 ← 382	Activation	0.164	0.612	0.216	0.007	OFF/ON
5580 ← 8613	Indirect	3.021	0.133	-0.819	0.019	OFF/ON
5604 ← 5894	Activation	0.160	0.183	0.273	0.041	OFF/ON
5321 ← 5595	Activation	0.356	0.535	-0.285	0.052	OFF/ON
382 ← 5880	Activation	0.302	0.455	1.314	0.002	OFF/ON
5580 ← 382	Directed path	-0.073	0.688	0.156	0.005	OFF/ON
382 ← 1399	Directed path	0.144	0.414	0.970	0.001	OFF/ON
7454 ← 5338	Directed path	0.111	0.358	1.070	0.002	OFF/ON
10093 ← 382	Directed path	0.269	0.252	0.498	0.001	OFF/ON
7454 ← 5880	String evidence	0.109	0.621	0.864	0.077	OFF/OFF
5581 ← 5595	String evidence	0.001	0.978	0.049	0.001	OFF/ON
23396 ← 5880	Activation	1.207	0.000	0.079	0.708	ON/OFF
5581 ← 8613	Indirect	0.953	0.000	0.002	0.921	ON/OFF
10093 ← 5880	Directed path	1.044	0.000	0.365	0.397	ON/OFF
8613 ← 1399	Directed path	-0.024	0.027	-0.093	0.225	ON/OFF
8936 ← 1794	Directed path	1.819	0.008	-1.627	0.608	ON/OFF
5580 ← 5338	String evidence	0.417	0.004	0.258	0.222	ON/OFF

**Figure 3:**
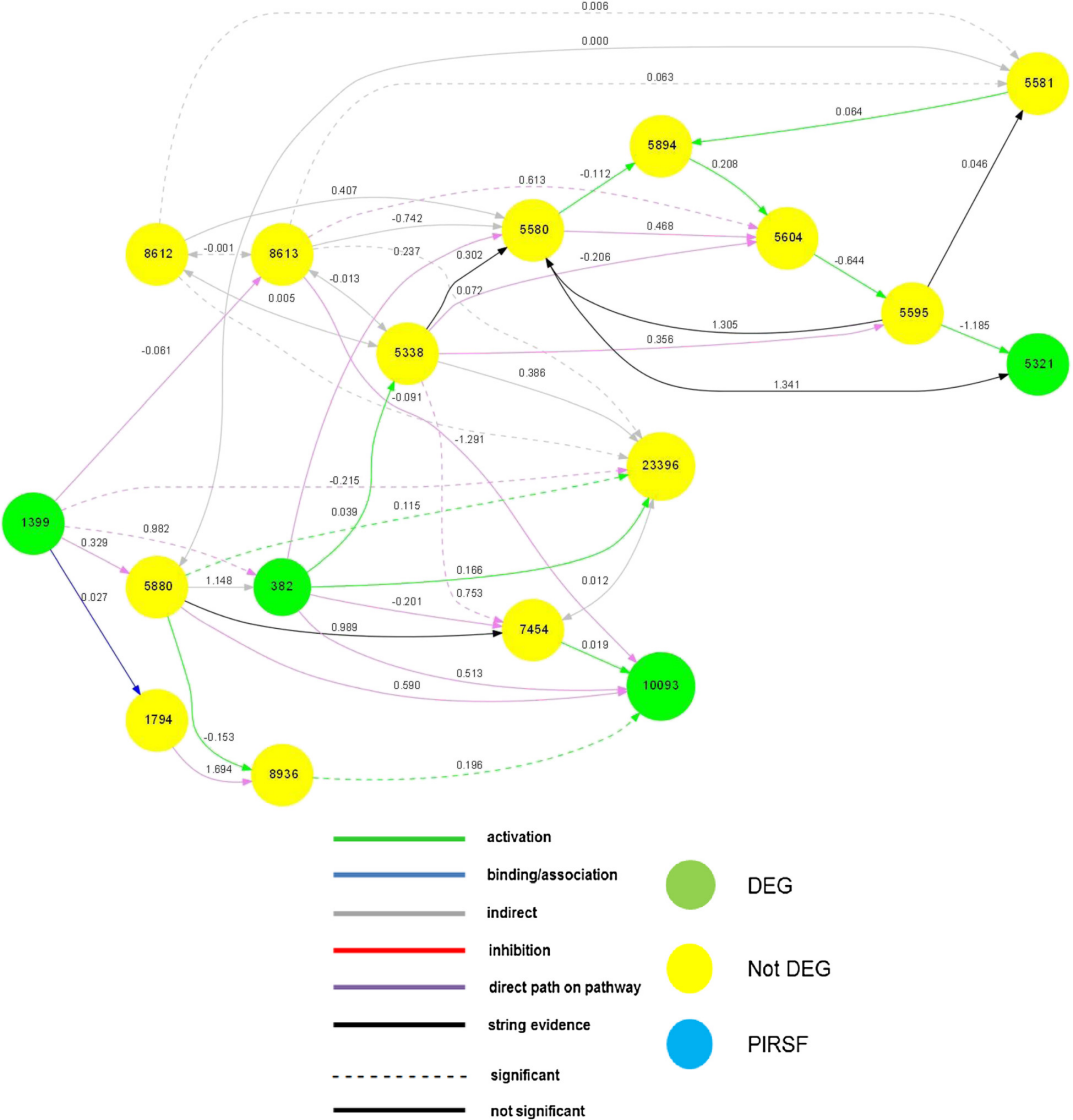
**Final model of Fc gamma R-mediated phagocytosis pathway.** The graph displays which nodes and edges are different between the disease/control groups. The nodes (with “Entrez” name) or edges are mapped with different colours according to up/down expression or regulation, respectively.

The comparison with KEGG edges showed that in the model pathway, 22 edges were not active (off) in the MS patients and 18 in controls. In contrast, the edges activated (on) were 6 and 10 in MS patients and control subjects respectively (cf. Table 6). The Figure 3 illustrates the pathway model and the links found statistically significant between the MS/control groups.

## Discussion

This work illustrated a new procedure based on Structural Equation Modeling (SEM) to discover and compare perturbed pathway-modules, similar to that proposed by [12]. Unlike these authors, new pathway analysis (SPIA) and new model generation, based on mixed graph theory and principles of network biology, were added. Starting from the significant dysregulated pathways, a model for each pathway was generated, that allowed both to verify how DEGs were connected between them in a causal structure and to improve the model interpretation by grouping not DEGs in PIR superfamilies. To note that the initial model is not inferred by data, because connections among genes represent the biological knowledge enclosed in the pathway and in PIRSFs. The use of SEM proved to be very versatile in the downstream analysis of microarray data. It was used to: 1) test and improve the generated models (this could be a useful way to overcome the limitation of accuracy relative to the public pathway database); 2) verify the overall differences between groups, and 3) individuate differential expression level of one gene and differential connection between two genes.

Our procedure was tested on two experiments of gene expression microarray data finalized to unravel the biological mechanisms that allowed us to explain the differences between yes/no mutant groups and disease/healthy groups. Starting from the output of SPIA, the model generation procedure, illustrated in this paper, was applied. The first step was to obtain a subgraph for each perturbed pathway containing only the genes present in the microarray. Then, the connections between DEGs through shortest paths were found, and finally not DEGs were grouped in PIRSFs. The models generated were tested and improved using an integrate approach based on the combination between SEM and other type of evidences as explicated in the STRING database. Once a good model has been determined, a two-groups SEM analysis has been performed to unveil significant differences between groups. Studying each link present in the final perturbed model, we hypothesized which connections could be altered. For example in FTLD-U experiment, we found the connection between SHANK2 and PSD-95, and in MS we hypothesized that the genes IL-1b, ARF6 and CRKL could be involved in a same pathological mechanism. These results confirmed and could be able to elucidate the mechanisms that lead to the pathogenesis and progression of the observed diseases.

The framework illustrated, being a composition of different methods, could be easily adapted to new solutions. The idea is to have a general and modular framework where different other methods could be taken in consideration in each step of the pipeline.

First, alternative ways to select DEGs and perturbed pathways could be considered. In [59] a unified framework was proposed to jointly find significant perturbed pathways and DEGs by sparse Linear Discriminant Analysis (sLDA). Other methods for identifying DEGs and differential connections, based on Graphical Gaussian Modeling (GGM), are the following: a first one generates networks directly from very high dimensional data, determining the pattern of zeros in the inverse covariance matrix [60]; a second one defines Bayesian networks (directed acyclic graph: DAGs) on a structure of dependence derived from external resources [61]; the last one derives DAGs from external resources converting them into undirected cycle-free graphs [62]. An additional approach for conducting a differential analysis of networks directly from data by measuring gene association/interaction with connectivity scores, based on Partial Least Squares (PLS), was suggested by [63].

Second, we used PCA to create new observed composite variables that represent PIRSFs. Another plan could be to take advantage of the potentiality of SEM in the creation of latent variables as proposed by [64] in transcriptional regulation of protein-DNA interactions. A valid alternative to the modification indices of SEM could be the PC-algorithm [65], that allow to infer causal information from data. The idea in this case is to fix an initial model, provided by the perturbed pathways model, and then use the PC-algorithm to add new links supervised by STRING knowledge. Lastly, since in SEM multigroup analysis numerous hypothesis are tested, multiple testing control procedures using the method of [66] can be desirable.

## Conclusions

The pipeline proposed introduces in the analysis of gene expression data the main principles that govern biological networks as well described by [14]. Otherwise from reverse-engineering gene regulatory networks, that build networks directly from data, our initial models are obtained by a biological curated pathway database (KEGG) and then modified on the basis of the knowledge provided by another database (STRING). The principal evidence is that the manifestation of a particular phenotype depends on the interactions existing among many causal agents. This was obtained taking in consideration not only DEGs but also how genes interact. The pipeline has been validated on two expression datasets. In both the cases, we tested the models, improved them and individuated the gene expression levels and the connections that were perturbed and that could justify the different phenotypes observed. The results were satisfactory and strongly coherent with experimental findings available in literature, considering that most of the genes in the model are known to characterize the phenomena analyzed and that links perturbed were previously connected to the progression of the diseases.

## Competing interests

The authors declare that they have no competing interests.

## Authors’ contribution

DP defined the procedure to generate models. MG supervised the work, in particular the statistical part, and together with DP, developed the procedure to test the models. DP and MG wrote jointly the paper. All the authors read and approved the final manuscript.

## Supplementary Material

Additional file 1 R codes for a demonstration of the procedure for the Multiple Sclerosis (MS) example. Click here for file
